# 3D-printed shadow masks for micro-patterned electrodes†

**DOI:** 10.1039/d4ra06298a

**Published:** 2024-10-29

**Authors:** Chanwook Cha, Eunhwa Jo, Yeongjun Kim, Andrew Jaeyong Choi, Koohee Han

**Affiliations:** a Department of Chemical Engineering, Kyungpook National University Daegu Republic of Korea han.koohee@knu.ac.kr; b School of Computing, Dept. of AI-SW, Gachon University 1342 Seongnam-daero, Sujeong-gu Seongnam 13306 Republic of Korea andrewjchoi@gachon.ac.kr

## Abstract

Microfabrication is critical to the advancement of lab-on-chip devices by enabling the creation of high-precision, complex electrode structures. Traditional photolithography, commonly used to fabricate micro-patterned electrodes, involves complex and multi-step processes that can be costly and time-consuming. In this research, we present a method using 3D-printed shadow masks for electrode fabrication, offering a simpler, cost-effective alternative to traditional methods. Specifically, by leveraging a fused deposition modeling 3D printer, we demonstrate that 3D-printed shadow masks streamline rapid prototyping of micro-patterned electrodes with a range of designs, from simple lines to complex patterns. To assess the lab-on-chip functionality of the electrodes fabricated from 3D-printed shadow masks, we investigate electric field-driven assembly of microparticles in the electrodes. The micro-patterned designs of the electrodes remotely guide the assembly patterns, resulting in the formation of well-defined, multiple chains and anisotropic structures. These results suggest that 3D-printed shadow masks not only simplify the fabrication process, but also maintain the precision required for advanced lab-on-chip applications. The proposed method could pave the way for more accessible and scalable manufacturing of the complex micro-patterned electrodes.

## Abstract 2

Streamlined production of micro-patterned electrodes using 3D-printed shadow masks provides a faster, cost-effective solution for rapid prototyping and iterative design, supporting the advancement of diverse lab-on-chip applications.

## Graphical abstract

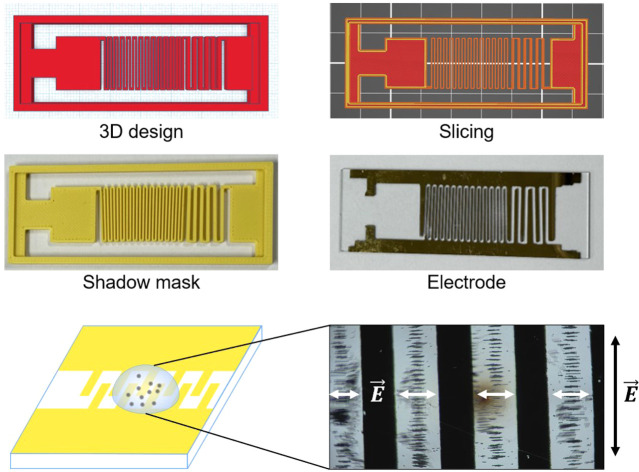

## 1 Introduction

Microfabrication plays a crucial role in the development of advanced lab-on-chip devices.^1–4^ Advancements in microfabrication technology have allowed the generation of high-precision and complex structures essential for the miniaturization of lab-on-chip devices.^5–7^ Especially, significant effort has been made to fabricate micro-patterned electrodes that enable greater design flexibility and enhanced device performance.^8–11^ The advanced lab-on-chip devices hold promise for innovative applications in a wide range of areas, including assembly,^12,13^ sensing,^14,15^ transport,^16,17^ diagnostics,^18,19^ and robotics.^20,21^

Photolithography has been widely used for fabricating micro-patterned electrodes.^22,23^ This approach involves the use of photoresists and multiple intricate steps such as coating, exposure, etching, and removal, resulting in a complex process.^24^ While the photolithography process ensures high precision down to nanometer levels, micro-patterned electrodes in lab-on-chip devices often do not require such an extreme level of precision in their patterns.^25^ Therefore, there is a promising opportunity to simplify the fabrication process of micro-patterned electrodes that could potentially save the cost and time.^26^

The most critical step in the fabrication process is to selectively deposit target metals onto the substrate surface.^27^ One facial method of the selective metal deposition is to use shadow masks.^28^ For example, during the process of metal vapor deposition, the shadow masks placed over the substrate surface effectively block the metal vapor, allowing the selective deposition of the metal only in the unshaded areas. The most common type of shadow mask is made of stainless steel because of its ease of production and durability in repeated use.^29^ However, stainless steel shadow masks have limitation in producing complicated patterns for micro-patterned electrodes.

Recent advancements in the 3D printing technology have shown the potential not only to create shadow masks capable of producing complex electrode patterns, but also to simplify the fabrication process.^30–32^ Specifically, the layer-by-layer additive manufacturing process minimizes the risk of distortion in the *x*, *y*, and *z* directions, thereby ensuring high accuracy in forming complex patterns.^33,34^ Also, modern 3D printing technology offers significant advantages over traditional methods, including lower costs, greater design flexibility, and easier usability.^35,36^

In this research, we propose an approach for fabricating micro-patterned electrodes through the use of 3D-printed shadow masks. By using the advantages of 3D printing, this research aims to simplify the fabrication process and enhance the adaptability of electrode designs. As an example, we have demonstrated the capability of 3D-printed shadow masks to manufacture micro-patterned electrodes with a range of designs, from simple lines to complex patterns. To validate the lab-on-chip functionality of the fabricated electrodes, we have performed electric field-driven assembly of the microparticles and have shown how the micro-patterned electrode designs affect the assembly patterns. The proposed approach enables faster, cheaper, and streamlined prototyping of electrodes, potentially contributing to the development of diverse lab-on-chip devices.

## 2 Materials and methods

### 2.1 Fabrication process of micro-patterned electrodes

**Fig. 1:**
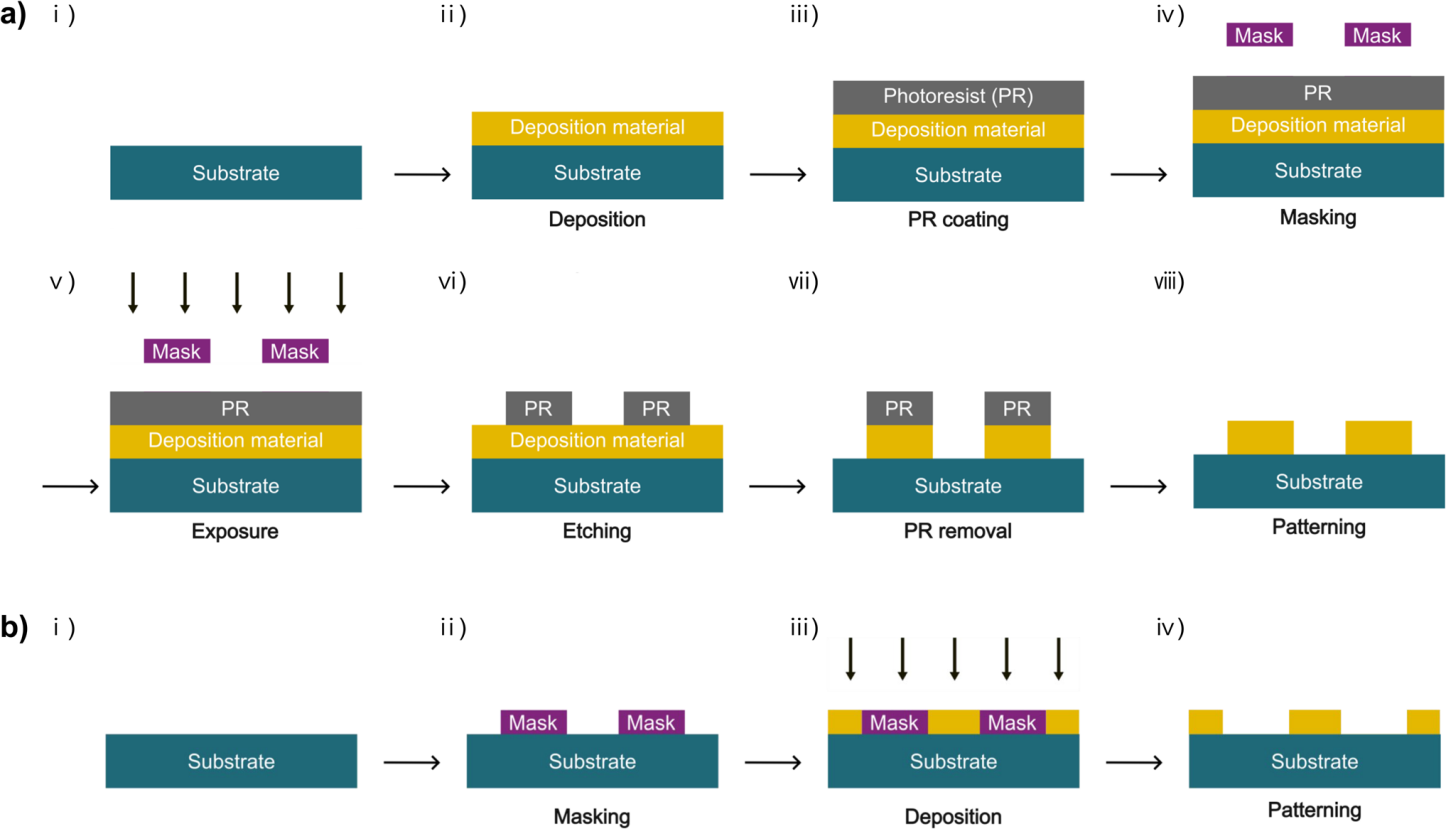
Comparison of using photolithography and 3D-printed shadow masks in the fabrication of patterned electrodes. (a) Schematic illustration of the fabrication steps for patterned electrodes using photolithography, consisting of (i) substrate preparation, (ii) deposition of conductive materials, (iii) coating with positive photoresist (PR), (iv) masking the PR with a photomask, (v) exposure of light through the patterned photomask to decompose the positive PR areas in the exposed areas, (vi) etching of the deposited conductive material in the exposed areas, (vii) removal of the remaining PR, and (viii) formation of the patterned electrode. (b) Schematic illustration of the fabrication steps for patterned electrodes using 3D-printed shadow masks, consisting of (i) substrate preparation, (ii) masking the substrate with a shadow mask, (iii) deposition of conductive materials through the shadow mask, and (iv) removal of the shadow mask to form the patterned electrode. This approach eliminates the need for PR coating, removal, and etching steps, thereby simplifying the workflow and enabling rapid production.

The schematics in Fig. 1 illustrate the fabrication process of the micro-patterned electrodes using conventional photolithography (Fig. 1a) and a 3D-printed shadow mask (Fig. 1b). The fabrication steps using photolithography are as follows: a conductive material, such as Cu or Au, is deposited onto a target substrate (Fig. 1a, i → ii); the deposited material is then uniformly coated with a positive photoresist that decomposes when exposed to light (Fig. 1a, iii); after positioning a photomask with desired patterns over the photoresist (Fig. 1a, iv), light selectively exposes the photoresist through the patterned photomask (Fig. 1a, v); the positive photoresist decomposes only in the exposed areas, leaving the photoresist on the masked areas (Fig. 1a, vi); an etching process removes the deposited material in the decomposed areas (Fig. 1a, vii); the removal of the remaining photoresist yields a micro-patterned electrode that matches the mask pattern (Fig. 1a, viii). The fabrication steps using a 3D-printed shadow mask are simpler than those involving photoresist: a 3D-printed patterned shadow mask is placed onto the substrate (Fig. 1b, i → ii); the conductive material is selectively deposited only to the areas not covered by the mask while leaving the masked areas undeposited (Fig. 1b, ii → iii); once the mask is removed, a micro-patterned electrode is formed that matches the inverse of the mask pattern (Fig. 1b, iv).

### 2.2 3D printer

A fused deposition modeling (FDM) 3D printer (Original Prusa MK4, Prusa Research) was utilized for this study. The filament used was a 1.75 mm diameter, matte polylactic acid (PLA) filament (high-strength PLA+, Sting3D). While various thermoplastic filaments are available for FDM 3D printing (see ESI†), PLA was selected for shadow mask fabrication due to its ease of process control, minimizing the need for complex parameter optimization (Table S1†). A 0.40 mm nozzle was employed for printing. The design for the 3D printing was created using web-based computer-aided design (CAD) software (Tinkercad, Autodesk Inc.). The 3D-printed shadow mask design consisted of a 2.0 mm thick shadow mask part and a 1.0 mm thick slide glass fix part. The shadow mask thickness was optimized to 2.0 mm to balance thermal deformation and shadow effects during metal evaporation, ensuring both stability and precision in metal patterning (Table S2†). The slicing was done using a 3D printing slicer (PrusaSlicer 2.8.0, Prusa Research) to generate the G-code. Here, the G-code is a programming code that instructs the 3D printer on how to move the nozzle and extruder, at what speed, and along which path to build the object layer by layer. The G-code was then loaded into the 3D printer to produce the 3D printed objects.

### 2.3 Electron beam evaporation

The micro-patterned electrodes were fabricated through electron beam (e-beam) evaporation, depositing thin metal films onto a slide glass substrate (75 mm × 25 mm × 1 mm, Marienfeld) using 3D-printed shadow masks (Fig. S1†). Detailed step-by-step procedures for electrode fabrication using 3D-printed shadow masks and e-beam evaporation are provided in the ESI.† Briefly, the slide glass was placed into the slide glass fix part of the 3D-printed shadow mask, securely attached, and then mounted inside an e-beam evaporator (KVE-2000, Korea Vacuum Tech Co.) equipped with a 7.1 kW e-Gun for metal deposition. Once mounted inside the vacuum chamber of the e-beam evaporator, a mechanical pump reduced the pressure to 3.0 × 10^−2^ torr within approximately 10 minutes, followed by a turbo pump achieving a high vacuum of 5.0 × 10^−6^ torr in about 1 hour. After reaching high vacuum, a 10 nm Cr adhesive layer was deposited at 4 ± 2% power of the e-Gun for 100 seconds at a rate of 1 Å s^−1^, followed by a 40 nm Au electrode layer deposited at 12 ± 2% power of the e-Gun for 400 seconds at a rate of 1 Å s^−1^. It took about 1 minute to gradually increase the power to achieve the desired deposition rate, and another minute to gradually reduce the power after deposition was complete. Metal deposition was confined to the areas of the slide glass not covered by the shadow mask, accurately reflecting the mask's design. In addition to conventional glass substrates, the e-beam evaporation process using 3D-printed shadow masks is compatible with various types of substrates, including a thermoplastic polyurethane (TPU) substrate (Fig. S2†).

### 2.4 Experimental setup

A 2.4 vol% colloidal solution containing 10 μm polystyrene particles (Yuanbiotech) was diluted with deionized water at a 1 : 10 volume ratio and then applied to the electrodes. An alternating current (AC) electric field at 100 kHz was applied using a function generator (DZ1022Z, Rigol) to remotely control the dynamic behavior of polystyrene particles. The 100 kHz frequency was selected to promote particle chaining behavior driven by dominant dipole–dipole interactions.^37,38^ The function generated was connected to an amplifier (WMA-300, Falco Systems) to ensure sufficient interaction strength, as lower amplitudes slow the chaining process.^38,39^ The assembly of the particles was observed in real time using a charge-coupled device (CCD) camera (DP28, Olympus) connected to an optical microscope (BX53, Olympus).

## 3 Results and discussion

### 3.1 3D design to patterned electrodes

**Fig. 2:**
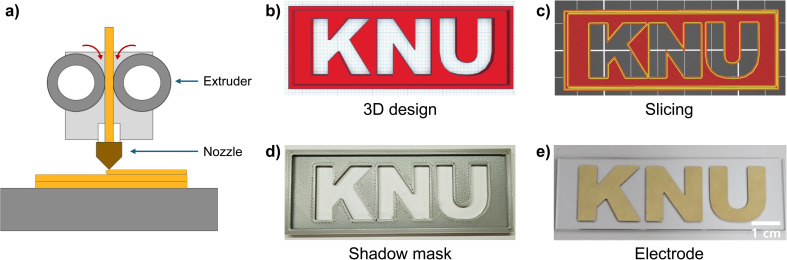
The use of a fused deposition modeling (FDM) 3D printer for creating shadow masks from 3D design to patterned electrodes. (a) Schematic illustration of the operating principles of the FDM 3D printer. The 3D printer nozzle heats the filament, and the extruder deposits it layer by layer. (b) 3D design of a shadow mask using computer-aided design (CAD) software. (c) Slicing the 3D design into multiple thin layers using slicing software, defining the printing path and adjusting various printer settings such as layer height, print speed, and nozzle temperature. (d) A shadow mask fabricated with the FDM 3D printer. (e) Resulting patterned electrode through selective metal deposition using the shadow mask.

Among the various types of 3D printers, we have used an FDM 3D printer to create the shadow masks and utilized them for the fabrication of micro-patterned electrodes as shown in Fig. 2. In FDM, the 3D printer nozzle heats a thermoplastic material and the extruder dispenses it layer by layer (Fig. 2a). FDM offers the benefits of low initial investment costs and easy operation/maintenance.^40,41^ Moreover, the FDM 3D printer facilitates the quick and straightforward design of various shapes, enabling rapid verification of the results.^42^ The process of fabricating micro-patterned electrodes using the FDM 3D printer is as follows. First, the desired pattern of the shadow mask is designed using CAD software (Fig. 2b). The shadow mask design is then sliced into thin layers, with the printing path coordinated along the *x*, *y*, and *z* axes using slicing software (Fig. 2c). The coordinated information guides the movements of the 3D printer to deposit the filament material layer by layer in the desired pattern. As the nozzle moves, the extruded filament cools and solidifies, forming the 3D-printed shadow mask (Fig. 2d). After placing the shadow mask on a substrate, such as slide glass, the conductive material is deposited through the metal evaporation method. Specifically, we use e-beam evaporation to deposit conductive materials like Cr, Au, and Cu. The 3D-printed shadow mask blocks the evaporated materials during the metal evaporation, ensuring the deposition only in the exposed areas. Once the shadow mask is removed, the desired electrode pattern is formed, reflecting the design of the shadow mask (Fig. 2e). Overall, employing a 3D-printed shadow mask for fabricating the micro-patterned electrodes simplifies the design process and expedites prototype production. Additionally, the precise control of the 3D printer nozzle in the *x* and *y* directions supports the creation of designs with angular and rounded shapes.

### 3.2 3D-printed shadow masks for micro-patterned electrodes

**Fig. 3:**
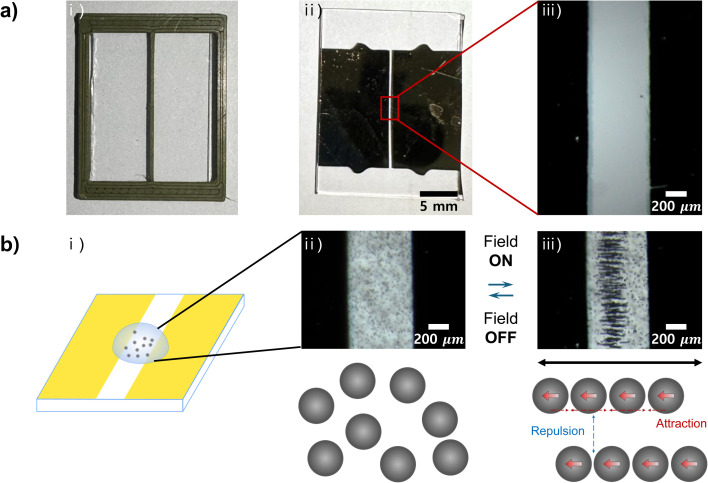
A micro-patterned electrode with a simple line. (a) Fabrication of a patterned electrode using a shadow mask with a simple line design. (i) Shadow mask produced with the FDM 3D printer, (ii) resulting patterned electrode created through selective metal deposition using the simple line shadow mask, and (iii) enlarged view of the simple line electrode, revealing smooth gold deposition on the substrate. (b) Electric field-driven assembly of polystyrene microparticles in the simple line electrode. (i) Schematic illustration of the experimental setup for the electric field-driven assembly. The yellow colors represent the Au electrodes. A drop of the aqueous solution containing 10 μm polystyrene particles is placed on the electrodes. (ii) Electric field-off state, showing dispersed particles in the aqueous solution. (iii) Electric field-on state, showing the assembly of multi-particle chains aligned in the direction of applied field between the Au electrodes. An alternating current (AC) electric field of 100 kHz and 1 V_pp_ is applied across the electrodes with a 0.5 mm gap. The direction of the global electric field is indicated by black color.

As an initial example, we fabricated a simple line patterned electrode by using the method described above (Fig. S3,† top). The steps are essentially the same, but the design is much simpler (Fig. 3a). After creating a shadow mask with a simple line design of 0.5 mm width (Fig. 3a, i), we used it to generate an electrode coated with 40 nm of Au, with a single line spaced 0.5 mm apart (Fig. 3a, ii). The enlarged view of the electrode shows that the areas covered by the shadow mask are free of Au, while the uncovered areas are well-deposited with Au (Fig. 3a, iii). In order to confirm whether the electrode functions properly, we observed the assembly behavior of colloidal particles under the application of an electric field in the electrode (Fig. 3b). A colloidal solution was prepared by mixing 10 μm diameter polystyrene particles with water, and a drop of the solution was placed on the electrode (Fig. 3b, i). An AC electric field of 100 kHz and 1 V_pp_ was applied to the particles through the electrode.

Under the application of the AC electric field, the polystyrene particles polarize and develop an induced dipole in the direction of the field due to the difference in dielectric constant between the particles and water.^43^ Once polarized, the particles with induced dipole interact with each other through dipole–dipole interactions. For simplicity, each dipole can be approximated as a point dipole.^44^ The external field in the single line electrode polarizes the particles to have a point dipole with the same magnitude and direction.^45,46^ In this case, the dipole–dipole interactions can be simplified and calculated based on the following equation:^47^1
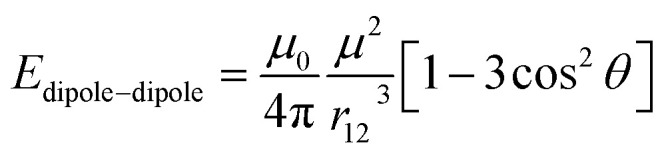
where *μ*_0_ represents the permeability of free space, *μ* is the magnitude of the dipole of the particles, *θ* denotes the angle between a dipole and the line connecting two neighboring dipoles, and *r*_12_ is the center-to-center distance between the dipoles.

The field-induced dipole–dipole interactions drove the assembly of the particles (Fig. 3b, ii → iii). When the electric field was turned on, the particles assembled into multiple linear chains (Fig. 3b, iii). The experimental result agrees well with eqn (1), as the dipole–dipole interaction energy, *E*_dipole–dipole_, decreases as *θ* approaches 0°. In other words, the dipole–dipole interaction energy between neighboring particles is minimized by achieving a head-to-tail configuration that aligns with the direction of the external field. On the other hand, the assembled linear chains repel each other when placed side-by-side, as the dipole–dipole interaction energy increases as *θ* approaches 180°. When the electric field was turned off, the chains disassemble due to the absence of dipole–dipole interactions (Fig. 3b, iii → ii). Consequently, the particles can reversibly assemble and disassemble by turning the field on and off, respectively (Fig. 3b, ii ↔ iii).

**Fig. 4:**
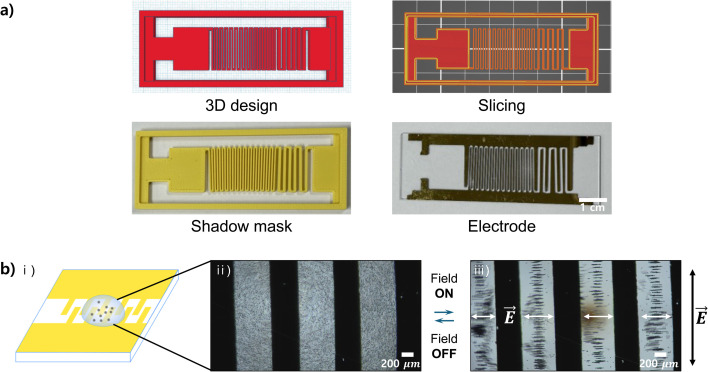
A micro-patterned interdigitated electrode. (a) Use of the FDM 3D printer for creating shadow masks with interdigitated shape from 3D design to the patterned electrode. (b) Electric field-driven assembly of polystyrene microparticles in the interdigitated electrode. (i) Schematic illustration of the experimental setup for the electric field-driven assembly. The yellow colors represent the Au electrodes. A drop of the aqueous solution containing 10 μm polystyrene particles is placed on the electrodes. (ii) Electric field-off state, showing dispersed particles in the solution. (iii) Electric field-on state, showing the assembly of multi-particle chains across several lines. A global AC electric field of 100 kHz and 2.5 V_pp_ is applied across the electrodes, forming local electric fields between the interlocking finger electrodes. The assembled chains are aligned in the direction of the local fields. The directions of the global and local electric fields are indicated by black and white colors, respectively.

The FDM 3D printer can be used to fabricate more complex electrode patterns. A representative example is interdigitated micro-patterned electrodes shown in Fig. 4 (and S3,† middle). The interdigitated shape is designed for use in devices such as organic field-effect transistors.^48–50^ It resembles two interlocking sets of fingers in a narrow space. This design allows for more patterns to be placed in a confined area, maximizing spatial efficiency and potentially enhancing electrical properties and response speed. In a similar manner to the process described above, the fabrication of interdigitated electrodes involved sequential steps of 3D design, slicing of the design, 3D printing of the shadow mask, and metal evaporation using the shadow mask for electrode production (Fig. 4a).

The assembly principle of particles in the simple line electrode can be extended to the assembly of particles in the interdigitated electrodes. After placing a solution of 10 μm polystyrene particles on the electrode, an AC electric field of 100 kHz and 2.5 V_pp_ was applied to observe the assembly behavior of the particles (Fig. 4b). This setup creates a global electric field between the main working and counter electrodes, similar to that in the simple line electrode. However, a key difference is that the interdigitated shape, resembling interlocking fingers, allows for the formation of multiple local electric fields between the “fingers,” perpendicular to the direction of the global electric field. Consequently, when the global field was turned on, the polystyrene particles assembled into multiple linear chains aligned with the local fields, perpendicular to the global field. Additionally, the interdigitated shape provided multiple distinct regions for assembly, enabling the polystyrene particles to assemble across several lines. We have also investigated the electrochemical characteristics of Au and Cu interdigitated electrodes by performing cyclic voltammetry (CV) analysis (Fig. S4†).^31,51^ The CV measurements reveal that Au electrodes exhibit supercapacitor-like behavior with primarily physical adsorption for charge storage, while Cu electrodes show clear redox behavior, indicating greater involvement of faradaic processes. This interdigitated configuration could be particularly useful in applications such as high-density sensor arrays, microfluidic control systems, and advanced diagnostic platforms.

**Fig. 5:**
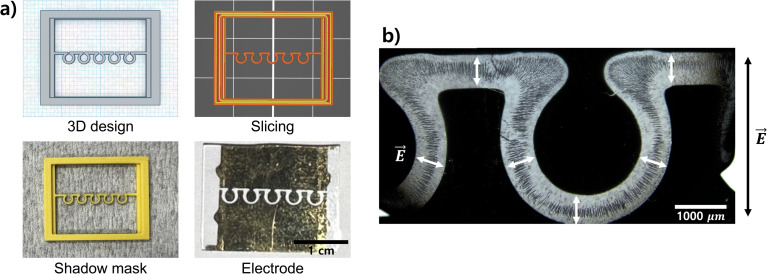
A micro-patterned electrode with a series of connected half-annulus shapes. (a) Use of the FDM 3D printer for creating shadow masks with connected half-annulus shapes from 3D design to the patterned electrode. (b) Electric field-driven assembly of polystyrene microparticles in the electrode with connected half-annulus shapes. A global AC electric field of 100 kHz and 2.5 V_pp_ is applied across the electrodes, generating local electric fields that vary in direction according to the contours of the half-annulus shapes. The assembled particle chains align in directionally-dependent configurations that follow the local field directions. The directions of the global and local electric fields are indicated by black and white colors, respectively.

A higher level of complexity in the shadow mask designs and the resulting patterned electrodes can be introduced by precisely controlling the 3D printer nozzle to create both angular and rounded shapes. We have demonstrated the example by designing a micro-patterned electrode with a series of connected half-annulus shapes (Fig. 5a and S3,† bottom). Similar to the interdigitated electrode, the connected half-annulus shapes rectify the global electric field to generate local electric fields in varying orientations at individual points. After placing a solution of 10 μm polystyrene particles on the electrode, an AC electric field of 100 kHz and 2.5 V_pp_ was applied to observe how the local electric fields affect the assembly patterns of the particles. The experimental result has demonstrated that the particles assembled in orientations that are precisely aligned with the local electric fields at various points (Fig. 5b). Specifically, the particles follow the contours of the electric field, organizing themselves in distinct, directionally-dependent configurations based on the varying orientations of the local electric fields within the structure. This alignment of the particles highlights the critical role of the local electric fields in directing particle assembly, resulting in well-defined, anisotropic patterns. Such configuration with varying orientations could be particularly useful in lab-on-chip applications requiring anisotropic properties, such as high-resolution microelectronics patterning, precision-targeted drug delivery systems, and directional sensing technologies.

## 4 Conclusions

In this research, we present a facile method for fabricating micro-patterned electrodes using the 3D-printed shadow masks. A comparison of the proposed approach and conventional techniques reveals trade-offs between resolution, cost, and accessibility (Table 1). Photolithography offers the highest resolution for the generation of micro-patterned electrodes, but involves a complex manufacturing process. Stainless steel shadow masks, produced through laser cutting, are faster and durable but have lower resolution. Laser cutting of stainless steels requires careful settings of operation parameters, particularly when generating overlapping patterns, which can lead to excessive heat buildup and dross formation. While both methods show promise in generating micro-patterned electrodes, their high initial investment costs may pose a hurdle for lab-scale operations. 3D-printed shadow masks provide a simple, fast, and low-cost alternative. However, a critical challenge with FDM 3D-printed shadow masks is that the printer nozzle limits the resolution to a few hundred micrometers. Despite this limitation, the method offers significant advantages by streamlining the manufacturing workflow and enhancing accessibility, as outlined below.

**Table 1:** Comparison of manufacturing techniques for micro-patterned electrodes

	Photolithography^2,22^	Stainless steel shadow mask^52^	FDM 3D-printed shadow mask^30,31,53^
Manufacturing process	Coating, exposure, etching, and removal of photoresists	Laser cutting of stainless steels	Additive manufacturing of thermoplastic materials
Process complexity	High	Medium	Low
Time	Long (2 ∼ 3 hours)	Short (∼1 hour)	Short (∼1 hour)
Initial investment cost	High to very high (around $100 000+)	High (around $100 000)	Low (around $1000)
Resolution	High to very high (tens of nanometers to a few micrometers)	Medium (tens of micrometers)	Low to medium (a few hundred micrometers)
Feasible patterns	Complex patterns	Simple channels^52^	Simple lines^30^
			Simple patterns^31^
			Complex patterns^53^
Advantages	High resolution	Fast production	Simple and fast production
		Durable structure	Easy and accessible
			Quick iterations
Disadvantages	High cost	Low resolution	Low resolution
	Complex process	Sensitive operation settings	Residual strings
		Thermal deformation	

One of the most significant advantages of our proposed method is its ability to expedite the prototyping process. The integration of 3D printing technology allows for the swift production of shadow masks and the subsequent fabrication of micro-patterned electrodes. For example, using an FDM 3D printer, all shadow mask designs demonstrated above can be produced in less than an hour. This rapid prototyping capability is crucial for the fast validation of various electrode designs. The ability for quick iterations and modifications enables more efficient optimization of electrode designs, thereby shortening the development cycle of new lab-on-chip devices.

Moreover, electrode fabrication using 3D-printed shadow masks offers clear advantages in scalability and adaptability. Conventional techniques often face limitations in adapting to various designs and substrates due to their complex top-down processes. In contrast, the bottom-up process of 3D printing allows for precise control over material deposition and layer formation in the *x*, *y*, and *z* axes, easily accommodating diverse patterns and substrates. This flexibility enhances the adaptability of electrode designs, providing tailored solutions for specific applications.^35^

Building on these principles, we successfully fabricate micro-patterned electrodes using 3D-printed shadow masks, ranging from simple lines to complex patterns, including interdigitated shapes and series of connected half-annulus forms. In response to the application of external electric fields, these electrodes remotely guide the assembly of microparticles into well-organized patterns dictated by the electrode designs. The ability of the micro-patterned electrodes to precisely control particle assembly opens up new possibilities for creating advanced, functional microstructures in lab-on-chip applications.

In summary, our research demonstrates that 3D-printed shadow masks offer significant benefits for micro-patterned electrode fabrication. The cost-effectiveness, scalability, adaptability, and rapid prototyping capabilities of this method position it as a promising alternative to conventional techniques. Consequently, this approach holds potential for the facile manufacturing of complex micro-patterned electrodes, enabling easy access to a wide range of lab-on-chip devices.

## Data availability

The data supporting this article have been included as part of the ESI.†

## Conflicts of interest

There are no conflicts to declare.

## Supplementary Material

RA-014-D4RA06298A-s001
