# Supplementary material for: 3D-printed shadow masks for micro-patterned electrodes
Source: RSC Adv. 2024 Oct 29;14(47):34586–93. doi: 10.1039/d4ra06298a (PMC11520313; doi:10.1039/d4ra06298a)
Supplement: RA-014-D4RA06298A-s001 [file RA-014-D4RA06298A-s001.pdf]

Supplementary Information (SI) for RSC Advances.
This journal is © The Royal Society of Chemistry 2024

Supplementary Information for

3D-Printed Shadow Masks for Micro-Patterned Electrodes

Chan Wook Cha[1,†], Eun Hwa Jo[1,†], Yeongjun Kim[1], Andrew Jaeyong Choi[2,*], Koohee Han[1,*]

[1]Department of Chemical Engineering, Kyungpook National University, Daegu, Republic of Korea
[2]School of Computing, Dept. of AI-SW, Gachon University, 1342 Seongnam-daero, Sujeong-gu, Seongnam 13306, Republic of Korea

[†]Contributed equally to this work
[*]Author to whom correspondence should be addressed.

[*]Corresponding author. E-mail: han.koohee@knu.ac.kr

Table S1. Different thermaplastic filament materials for FDM 3d-printed shadow masks

	Thermaplastic filament materials			
	**PLA**	**PETG**	**ASA**	**TPU**
**Structural stability**	Medium	Medium	Medium	Very low
**Cost** ($/kg)	29.99	29.99	35.38	63.99
**Ease of fabrication**	Easy	Normal	Hard	Hard
**Fabrication result**	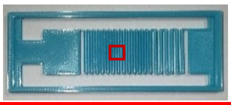	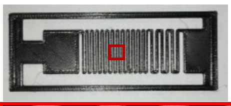	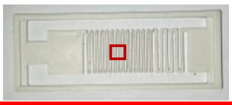	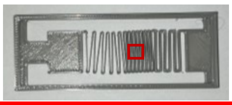
**Stringing effect**	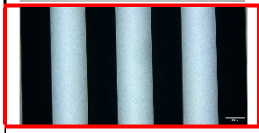<br>No	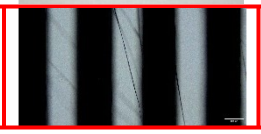<br>Yes	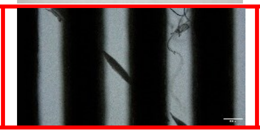<br>Yes	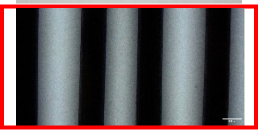<br>No

For FDM 3D printing, several thermoplastic filament materials can be used, including polylactic acid (PLA), polyethylene terephthalate glycol (PETG), acrylonitrile-styrene-acrylate (ASA), and thermoplastic polyurethane (TPU). Compared to other filaments, PLA offers easier process control, as adjusting the nozzle temperature is usually sufficient to optimize the 3D printing quality. In contrast, PETG and ASA require more precise tuning of multiple parameters, such as nozzle temperature, retraction time, and bed adhesion, to achieve optimal results. Without properly optimizing these settings, shadow masks fabricated from PETG and ASA often leave behind thin filament residues, which could potentially interfere with the metal vapor deposition. TPU is not suitable for shadow mask fabrication, as those made from TPU are too soft to retain the mask structure. For simplicity, we selected PLA filament to generate various shadow mask designs.

Table S2. Optimization of 3D-printed shadow mask thickness

	Shadow mask thickness					
	**0.5 mm**		**2.0 mm**		**5.0 mm**	
**CAD design**	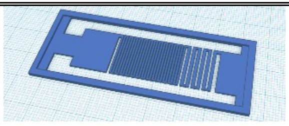		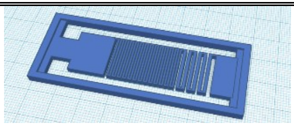		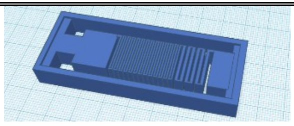	
	Thin and narrow patterns	Thick and wide patterns	Thin and narrow patterns	Thick and wide patterns	Thin and narrow patterns	Thick and wide patterns
**Deposition result**	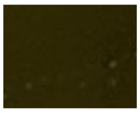 2.5 mm	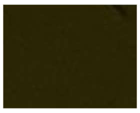 2.5 mm	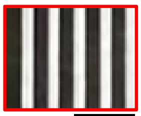 2.5 mm	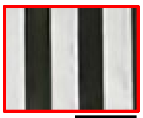 2.5 mm	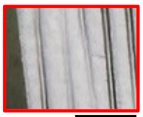 2.5 mm	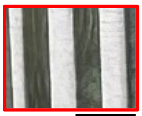 2.5 mm
**Error average**	-		9.200 %	12.716 %	86.110 %	15.006 %

The thickness of the 3D-printed shadow masks was determined by two key factors. The first is the thermal deformation of the masks during the metal evaporation process. For example, with a thickness of 0.5 mm, the low thermal resistance of the thin shadow masks results in sagging, preventing them from effectively shielding the design from the metal vapor. The second is the shadow effects that physically interfere with the diffusion of the metal vapor. For example, at 5.0 mm, the thick shadow masks hindered metal deposition, leading to significantly reduced coating, especially for narrow and thin patterns. To manage these trade-offs, the shadow mask thickness was optimized to 2.0 mm, minimizing both thermal deformation and shadow effects. The error average reflects the discrepancy between the CAD design and the actual deposition across two distinct regions: 1) thin and narrow patterns; 2) thick and wide patterns.

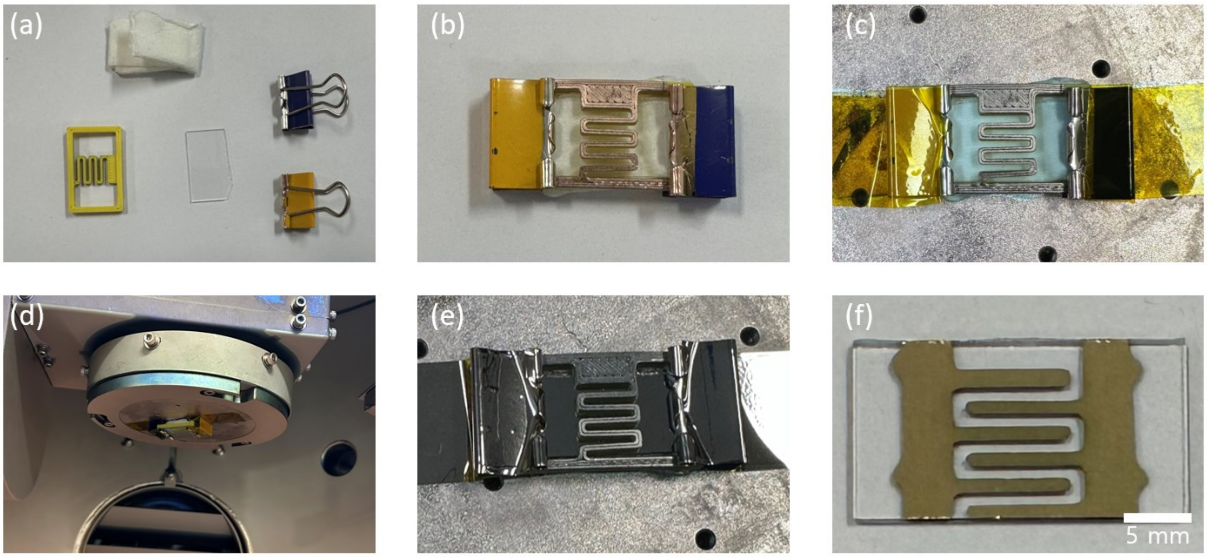

Figure S1. Step-by-step procedures for electrode fabrication using 3D-printed shadow masks and e-beam evaporation.

Figure S1 shows the detailed step-by-step procedures for fabricating micro-patterned electrodes via e-beam evaporation. First, the slide glass was cut to match the size of the 3D-printed shadow mask and cleaned sequentially in acetone, ethanol, and deionized water for 5 minutes each (**Fig. S1a**). Next, the slide glass was positioned in the designated holder of the shadow mask and securely fixed with clips, placing tissues between the glass slide and the shadow mask to minimize gaps and ensure a tight fit (**Fig. S1b**). The assembly was then attached to the e-beam substrate using polyimide tape (**Fig. S1c**), and the substrate was secured inside the vacuum chamber of the e-beam evaporator (**Fig. S1d**). Following metal evaporation, the target metal was deposited on all exposed surfaces (**Fig. S1e**), while the shadow mask effectively blocked deposition onto the glass substrate, resulting in the formation of the desired metal patterns (**Fig. S1f**).

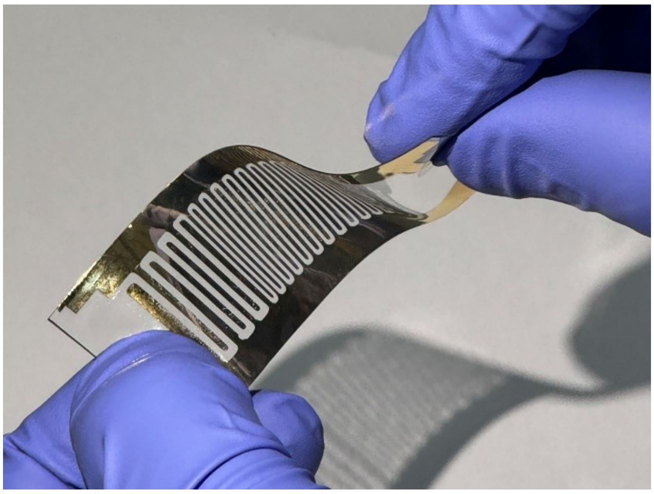

Figure S2. Interdigitated electrodes fabricated by depositing Au metal vapor through a 3D-printed shadow mask onto a TPU substrate

In addition to conventional glass slides, 3D-printed shadow masks are compatible with various types of substrates. For example, we demonstrated the fabrication of interdigitated electrodes on a thermoplastic polyurethane (TPU) substrate using a 3D-printed shadow mask (**Fig. S2**). The interdigitated electrodes on the TPU substrate accurately replicate the shadow mask design while preserving the overall flexibility of the TPU substrate.

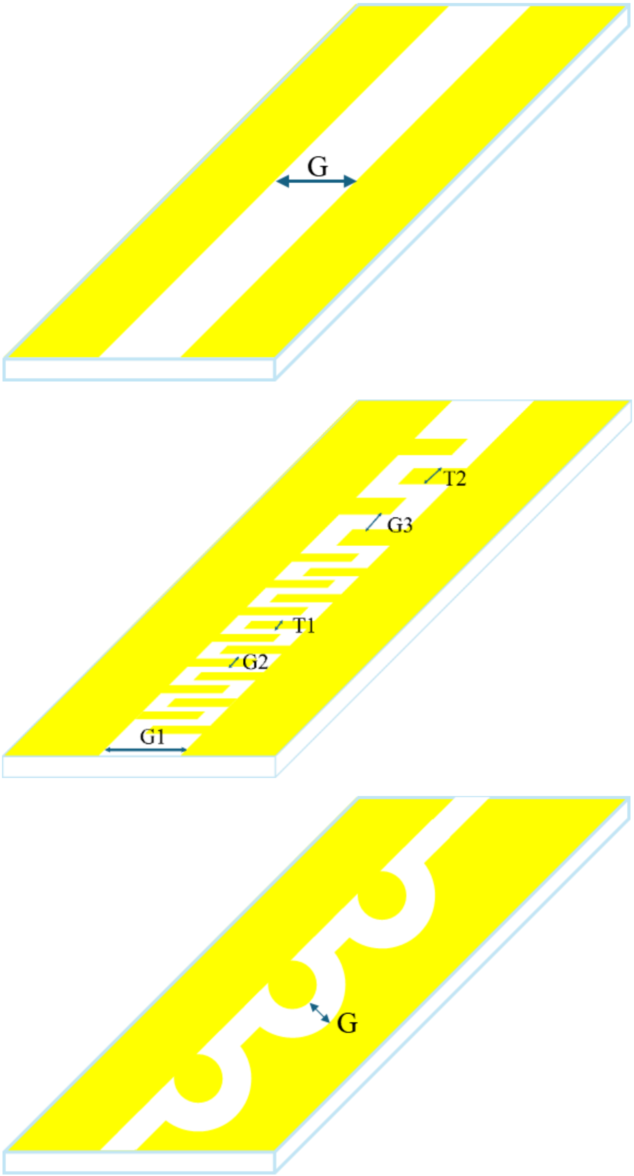

Figure S3. Schematic illustrations of the single line electrode (top), interdigitated electrode (middle), electrode with a series of connected half-annulus shapes (bottom).

Figure S3 illustrates three different designs of micro-patterned electrodes used in this research. The yellow areas represent the deposited Au electrodes. For the single-line electrode, the gap between the electrodes (G) was set to 0.5 mm (**Fig. S3, top**). The interdigitated electrode includes two different patterns of finger electrodes, where the gap between the electrodes (G1) was set to 1.7 mm (**Fig. S3, middle**). The gaps between the first finger electrodes (G2) were set to 0.5 mm, and the gaps between the second finger electrodes (G3) were set to 1.0 mm. The thickness of the first finger electrodes (T1) was set to 0.5 mm, while the thickness of the second finger electrodes (T2) was set to 1.0 mm. For the electrode with a series of connected half-annulus shapes, the gap between the electrodes (G) was set to 0.5 mm (**Fig. S3, bottom**).

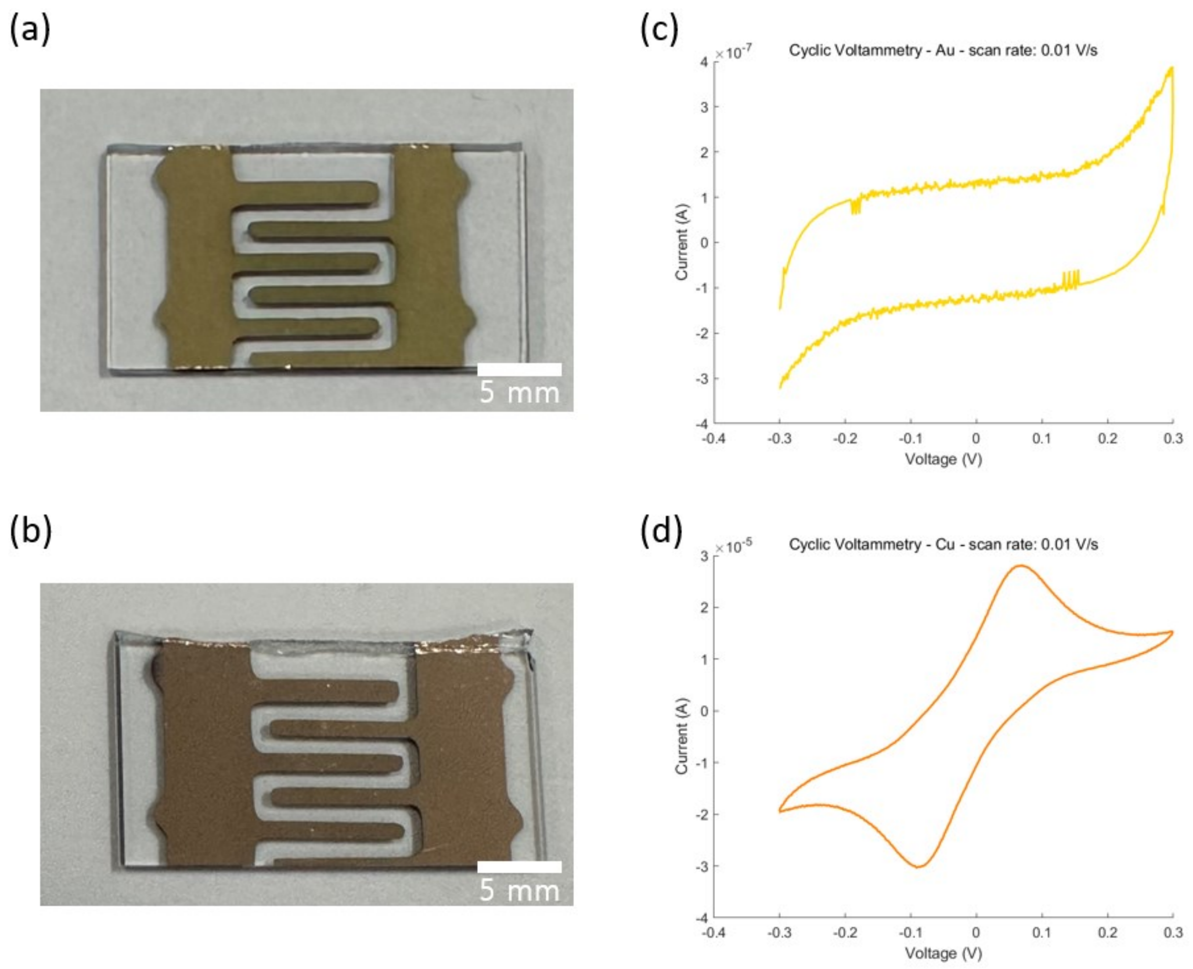

Figure S4. Cyclic voltammetry (CV) curves of interdigitated electrodes fabricated with gold (Au) and copper (Cu).

We investigated the electrochemical performance of micro-patterned electrodes using cyclic voltammetry (CV) analysis (**Fig. S4**). Two types of interdigitated electrodes, one with gold (Au) and the other with copper (Cu), were fabricated and compared (**Fig. S4a and S4b**). A gel electrolyte was prepared from a polyvinyl alcohol (PVA) and NaCl solution, in which 0.351 g of NaCl was dissolved in 20 g of deionized water, followed by the addition of 2 g of PVA (molecular weight ~126,000 g/mol) to form a homogeneous gel. The CV measurements were performed at a scan rate of 0.01 V/s.

The CV curves for the Au interdigitated electrodes exhibit a quasi-rectangular shape, with the central region displaying supercapacitor-like behavior (**Fig. S4c**). However, the absence of distinct oxidation/reduction peaks suggests that the charge storage mechanism is primarily driven by physical adsorption rather than faradaic processes. This quasi-rectangular shape can be attributed to the planar gold coating, which restricts the effective surface area for ion adsorption. Incorporating active materials such as activated carbon or carbon nanotubes could

increase the available ion adsorption sites, thereby enhancing charge storage capacity and improving overall supercapacitor performance.

In contrast, the CV curves for the Cu interdigitated electrodes exhibit a conventional “duck” shape, with clear oxidation and reduction peaks (**Fig. S4d**). Oxidation occurs around 0.08 V, corresponding to the formation of Cu^{2+} ions, while reduction takes place near -0.08 V, where Cu^{2+} ions is redeposited onto the electrode surface. These faradaic processes reflect typical redox behavior, suggesting that Cu electrodes operate through a different charge storage mechanism compared to Au, with greater involvement of chemical reactions.
